# Bis(acetato-κ*O*)bis­(2-pyridine­aldoxime-κ^2^ *N*,*N*′)cadmium

**DOI:** 10.1107/S1600536812031819

**Published:** 2012-07-18

**Authors:** Sadif A. Shirvan, Sara Haydari Dezfuli

**Affiliations:** a Department of Chemistry, Islamic Azad University, Omidieh Branch, Omidieh, Iran

## Abstract

In the title mol­ecule, [Cd(CH_3_COO)_2_(C_6_H_6_N_2_O)_2_], the Cd^II^ cation is *N*,*N*′-chelated by two 2-pyridine­aldoxime ligands and coordinated by two acetate anions in a distorted octa­hedral geometry. The hy­droxy groups of the 2-pyridine­aldoxime ligands link to the acetate anions *via* intra­molecular O—H⋯O hydrogen bonds. Weak inter­molecular C—H⋯O hydrogen bonds occur in the crystal.

## Related literature

For related structures, see: Abu-Youssef *et al.* (2010 ▶); Costa *et al.* (2009 ▶); Ha (2010 ▶); Konidaris *et al.* (2010 ▶); Korpi *et al.* (2005 ▶); Milios *et al.* (2004 ▶); Mukherjee *et al.*(2009 ▶); Torabi *et al.* (2005 ▶).
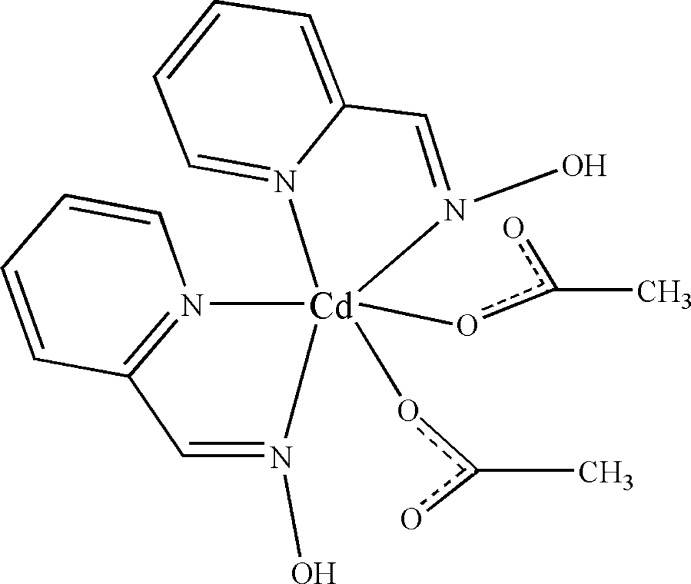


## Experimental

### 

#### Crystal data

[Cd(C_2_H_3_O_2_)_2_(C_6_H_6_N_2_O)_2_]
*M* *_r_* = 474.75
Triclinic, 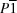

*a* = 8.7875 (6) Å
*b* = 9.0946 (6) Å
*c* = 13.8873 (11) Å
α = 100.837 (6)°
β = 97.994 (6)°
γ = 114.700 (5)°
*V* = 960.42 (12) Å^3^
*Z* = 2
Mo *K*α radiation
μ = 1.18 mm^−1^
*T* = 298 K
0.48 × 0.38 × 0.30 mm

#### Data collection

Bruker APEXII CCD area-detector diffractometer
Absorption correction: multi-scan (*SADABS*; Bruker, 2001 ▶) *T*_min_ = 0.621, *T*_max_ = 0.751
8165 measured reflections
3758 independent reflections
3236 reflections with *I* > 2σ(*I*)
*R*_int_ = 0.056

#### Refinement

*R*[*F* ^2^ > 2σ(*F* ^2^)] = 0.034
*wR*(*F* ^2^) = 0.084
*S* = 0.99
3758 reflections
252 parameters
2 restraints
H atoms treated by a mixture of independent and constrained refinement
Δρ_max_ = 0.64 e Å^−3^
Δρ_min_ = −0.96 e Å^−3^

### 

Data collection: *APEX2* (Bruker, 2007 ▶); cell refinement: *SAINT* (Bruker, 2007 ▶); data reduction: *SAINT*; program(s) used to solve structure: *SHELXS97* (Sheldrick, 2008 ▶); program(s) used to refine structure: *SHELXL97* (Sheldrick, 2008 ▶); molecular graphics: *SHELXTL* (Sheldrick, 2008 ▶); software used to prepare material for publication: *SHELXTL*.

## Supplementary Material

Crystal structure: contains datablock(s) I, global. DOI: 10.1107/S1600536812031819/xu5584sup1.cif

Structure factors: contains datablock(s) I. DOI: 10.1107/S1600536812031819/xu5584Isup2.hkl

Additional supplementary materials:  crystallographic information; 3D view; checkCIF report

## Figures and Tables

**Table 1:** Selected bond lengths (Å)

Cd1—N1	2.438 (3)
Cd1—N2	2.362 (3)
Cd1—N3	2.411 (3)
Cd1—N4	2.344 (3)
Cd1—O3	2.245 (3)
Cd1—O5	2.210 (3)

**Table 2:** Hydrogen-bond geometry (Å, °)

*D*—H⋯*A*	*D*—H	H⋯*A*	*D*⋯*A*	*D*—H⋯*A*
O1—H1*B*⋯O6	0.83 (2)	1.72 (1)	2.545 (4)	169 (4)
O2—H2*B*⋯O4	0.83 (5)	1.69 (5)	2.512 (5)	175 (6)
C6—H6*A*⋯O3^i^	0.93	2.52	3.381 (5)	153
C9—H9⋯O5^ii^	0.93	2.55	3.387 (6)	151
C12—H12*A*⋯O6^iii^	0.93	2.56	3.264 (6)	132

Symmetry codes: (i) 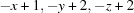; (ii) 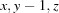; (iii) 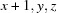.

**Fig. 1.**
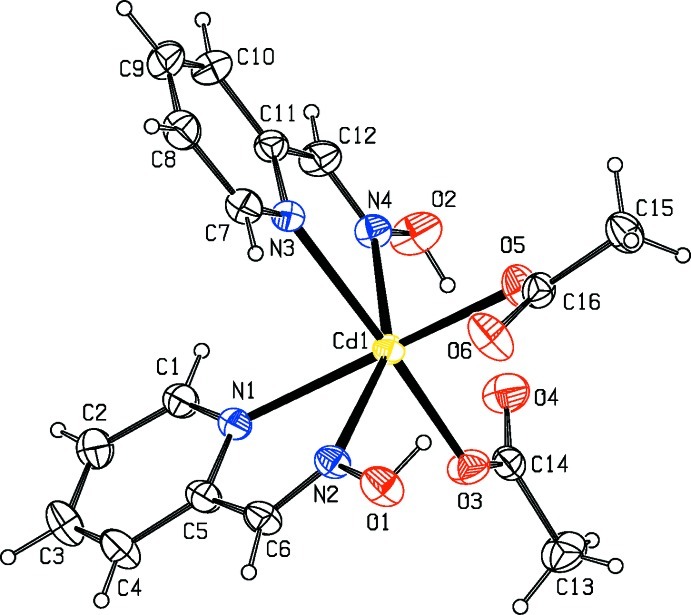
The molecular structure of the title molecule, with the atom-numbering scheme. Displacement ellipsoids are drawn at the 30% probability level.

**Fig. 2.**
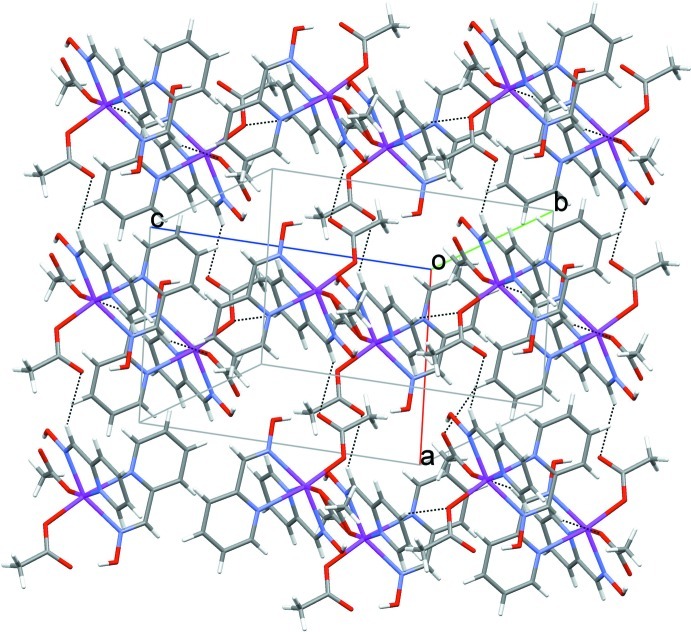
Unit-cell packing diagram for title compound.

## supplementary crystallographic information

### Comment

2-Pyridinealdoxime (pya), is a good bidentate ligand, and numerous complexes with pya have been prepared, such as that of manganese (Ha, 2010; Milios *et al.*, 2004), rhenium (Costa *et al.*, 2009), nickel (Mukherjee, *et al.*, 2009), silver (Abu-Youssef *et al.*, 2010), mercury (Torabi *et al.*, 2005), zinc (Konidaris *et al.*, 2010) and copper (Korpi *et al.*, 2005). Here, we report the synthesis and structure of the title compound.

In the molecule of the title compound, (Fig. 1), the Cd^II^ atom is six-coordinated in a distorted octahedral configurations by four N atoms from two 2-pyridinealdoxime ligands and two O atom from two acetate anions. The Cd—O and Cd—N bond lengths and angles are collected in Table 1.

In the crystal structure, intermolecular O—H···O and C—H···O hydrogen bonds form a three-dimensional network (Table 2 & Fig. 2).

### Experimental

A solution of 2-pyridinealdoxime (0.50 g, 4.0 mmol) in methanol (15 ml) was added to a solution of Cd(OAc)_2_.2H_2_O (0.54 g, 2.0 mmol) in methanol (15 ml) and the resulting colorless solution was stirred for 15 min at room temperature. This solution was left to evaporate slowly at room temperature. After one week, colorless prismatic crystals of the title compound were isolated (yield 0.69 g, 72.7%).

### Refinement

Hydroxyl H atoms were located in a difference Fourier map and refined isotropically. Other H atoms were positioned geometrically, with C—H = 0.93 and 0.96 Å and constrained to ride on their parent atoms with U_iso_(H) = 1.5U_eq_(C) for methyl H atoms and 1.2U_iso_(C) for the others.

### Crystal data

**Table:**

[Cd(C_2_H_3_O_2_)_2_(C_6_H_6_N_2_O)_2_]	*Z* = 2
*M**_r_* = 474.75	*F*(000) = 476
Triclinic, *P*1	*D*_x_ = 1.642 Mg m^−^^3^
Hall symbol: -P 1	Mo *K*α radiation, λ = 0.71073 Å
*a* = 8.7875 (6) Å	Cell parameters from 8165 reflections
*b* = 9.0946 (6) Å	θ = 1.5–26.0°
*c* = 13.8873 (11) Å	µ = 1.18 mm^−^^1^
α = 100.837 (6)°	*T* = 298 K
β = 97.994 (6)°	Prism, colorless
γ = 114.700 (5)°	0.48 × 0.38 × 0.30 mm
*V* = 960.42 (12) Å^3^	

### Data collection

**Table:**

Bruker APEXII CCD area-detector diffractometer	3758 independent reflections
Radiation source: fine-focus sealed tube	3236 reflections with *I* > 2σ(*I*)
Graphite monochromator	*R*_int_ = 0.056
ω scans	θ_max_ = 26.0°, θ_min_ = 1.5°
Absorption correction: multi-scan (*SADABS*; Bruker, 2001)	*h* = −10→10
*T*_min_ = 0.621, *T*_max_ = 0.751	*k* = −11→11
8165 measured reflections	*l* = −17→15

### Refinement

**Table:**

Refinement on *F*^2^	Primary atom site location: structure-invariant direct methods
Least-squares matrix: full	Secondary atom site location: difference Fourier map
*R*[*F*^2^ > 2σ(*F*^2^)] = 0.034	Hydrogen site location: inferred from neighbouring sites
*wR*(*F*^2^) = 0.084	H atoms treated by a mixture of independent and constrained refinement
*S* = 0.99	*w* = 1/[σ^2^(*F*_o_^2^) + (0.0491*P*)^2^] where *P* = (*F*_o_^2^ + 2*F*_c_^2^)/3
3758 reflections	(Δ/σ)_max_ = 0.010
252 parameters	Δρ_max_ = 0.64 e Å^−^^3^
2 restraints	Δρ_min_ = −0.96 e Å^−^^3^

### Special details

**Table:**

Geometry. All e.s.d.'s (except the e.s.d. in the dihedral angle between two l.s. planes) are estimated using the full covariance matrix. The cell e.s.d.'s are taken into account individually in the estimation of e.s.d.'s in distances, angles and torsion angles; correlations between e.s.d.'s in cell parameters are only used when they are defined by crystal symmetry. An approximate (isotropic) treatment of cell e.s.d.'s is used for estimating e.s.d.'s involving l.s. planes.
Refinement. Refinement of *F*^2^ against ALL reflections. The weighted *R*-factor *wR* and goodness of fit *S* are based on *F*^2^, conventional *R*-factors *R* are based on *F*, with *F* set to zero for negative *F*^2^. The threshold expression of *F*^2^ > σ(*F*^2^) is used only for calculating *R*-factors(gt) *etc*. and is not relevant to the choice of reflections for refinement. *R*-factors based on *F*^2^ are statistically about twice as large as those based on *F*, and *R*- factors based on ALL data will be even larger.

### Fractional atomic coordinates and isotropic or equivalent isotropic displacement parameters (Å^2^)

**Table:**

	*x*	*y*	*z*	*U*_iso_*/*U*_eq_	
C1	0.8192 (5)	0.7991 (5)	0.9158 (3)	0.0581 (9)	
H1	0.8759	0.8033	0.8639	0.070*	
C2	0.8947 (6)	0.7893 (6)	1.0068 (3)	0.0722 (12)	
H2	0.9992	0.7843	1.0152	0.087*	
C3	0.8151 (6)	0.7869 (7)	1.0838 (4)	0.0845 (14)	
H3	0.8653	0.7822	1.1460	0.101*	
C4	0.6590 (6)	0.7916 (7)	1.0692 (3)	0.0781 (13)	
H4	0.6029	0.7915	1.1213	0.094*	
C5	0.5877 (5)	0.7965 (5)	0.9757 (3)	0.0570 (9)	
C6	0.4177 (5)	0.7942 (5)	0.9549 (3)	0.0601 (9)	
H6A	0.3628	0.8031	1.0067	0.072*	
C7	0.3108 (5)	0.3972 (5)	0.6895 (3)	0.0564 (9)	
H7	0.2298	0.4142	0.7203	0.068*	
C8	0.2865 (6)	0.2340 (5)	0.6552 (3)	0.0672 (11)	
H8	0.1925	0.1439	0.6637	0.081*	
C9	0.4040 (6)	0.2086 (5)	0.6088 (4)	0.0731 (12)	
H9	0.3917	0.1006	0.5853	0.088*	
C10	0.5391 (6)	0.3433 (5)	0.5972 (3)	0.0686 (11)	
H10	0.6188	0.3277	0.5643	0.082*	
C11	0.5577 (4)	0.5027 (5)	0.6342 (3)	0.0508 (8)	
C12	0.7043 (5)	0.6520 (5)	0.6258 (3)	0.0649 (11)	
H12A	0.7859	0.6391	0.5939	0.078*	
C13	0.8125 (7)	1.3947 (5)	0.8418 (4)	0.0858 (14)	
H13A	0.8408	1.4150	0.9139	0.103*	
H13B	0.7162	1.4160	0.8218	0.103*	
H13C	0.9102	1.4682	0.8214	0.103*	
C14	0.7660 (5)	1.2150 (5)	0.7920 (3)	0.0560 (9)	
C15	0.0811 (5)	0.7410 (6)	0.4978 (3)	0.0698 (11)	
H15A	0.1029	0.6727	0.4453	0.084*	
H15B	0.1167	0.8513	0.4882	0.084*	
H15C	−0.0400	0.6904	0.4955	0.084*	
C16	0.1808 (5)	0.7544 (5)	0.5986 (3)	0.0540 (9)	
N1	0.6675 (4)	0.8026 (4)	0.8998 (2)	0.0509 (7)	
N2	0.3474 (4)	0.7801 (4)	0.8656 (2)	0.0520 (7)	
N3	0.4438 (3)	0.5309 (3)	0.6806 (2)	0.0459 (6)	
N4	0.7206 (4)	0.7966 (4)	0.6613 (2)	0.0519 (7)	
O1	0.1873 (4)	0.7749 (5)	0.8529 (2)	0.0717 (8)	
H1B	0.159 (5)	0.763 (5)	0.7912 (10)	0.058 (12)*	
O2	0.8603 (4)	0.9252 (4)	0.6470 (3)	0.0863 (10)	
H2B	0.864 (7)	1.016 (4)	0.675 (4)	0.11 (2)*	
O3	0.6409 (4)	1.1036 (3)	0.8110 (2)	0.0700 (8)	
O4	0.8568 (4)	1.1901 (4)	0.7372 (3)	0.0850 (9)	
O5	0.3410 (3)	0.8162 (4)	0.6126 (2)	0.0682 (7)	
O6	0.0997 (4)	0.7014 (5)	0.6609 (2)	0.0892 (10)	
Cd1	0.51653 (3)	0.82333 (3)	0.745759 (18)	0.04330 (10)	

### Atomic displacement parameters (Å^2^)

**Table:**

	*U*^11^	*U*^22^	*U*^33^	*U*^12^	*U*^13^	*U*^23^
C1	0.062 (2)	0.063 (2)	0.051 (2)	0.0298 (19)	0.0140 (17)	0.0144 (18)
C2	0.072 (3)	0.082 (3)	0.066 (3)	0.042 (2)	0.006 (2)	0.019 (2)
C3	0.088 (3)	0.109 (4)	0.055 (3)	0.044 (3)	0.002 (2)	0.032 (3)
C4	0.087 (3)	0.106 (4)	0.045 (2)	0.045 (3)	0.015 (2)	0.026 (2)
C5	0.064 (2)	0.061 (2)	0.0416 (19)	0.0260 (18)	0.0115 (16)	0.0115 (17)
C6	0.066 (2)	0.073 (3)	0.046 (2)	0.032 (2)	0.0223 (17)	0.0176 (18)
C7	0.0511 (19)	0.058 (2)	0.054 (2)	0.0191 (17)	0.0148 (16)	0.0152 (18)
C8	0.069 (2)	0.054 (2)	0.064 (3)	0.0150 (19)	0.012 (2)	0.019 (2)
C9	0.088 (3)	0.048 (2)	0.077 (3)	0.030 (2)	0.012 (2)	0.013 (2)
C10	0.076 (3)	0.062 (2)	0.074 (3)	0.040 (2)	0.023 (2)	0.009 (2)
C11	0.0512 (18)	0.055 (2)	0.0488 (19)	0.0270 (16)	0.0140 (15)	0.0122 (16)
C12	0.060 (2)	0.071 (3)	0.074 (3)	0.034 (2)	0.036 (2)	0.018 (2)
C13	0.098 (3)	0.047 (2)	0.095 (4)	0.020 (2)	0.022 (3)	0.010 (2)
C14	0.065 (2)	0.053 (2)	0.045 (2)	0.0239 (18)	0.0067 (17)	0.0140 (17)
C15	0.062 (2)	0.092 (3)	0.059 (2)	0.038 (2)	0.0087 (19)	0.024 (2)
C16	0.051 (2)	0.055 (2)	0.057 (2)	0.0275 (17)	0.0068 (16)	0.0141 (17)
N1	0.0543 (16)	0.0539 (17)	0.0436 (16)	0.0251 (14)	0.0106 (13)	0.0112 (13)
N2	0.0485 (15)	0.0629 (19)	0.0475 (17)	0.0252 (14)	0.0201 (13)	0.0163 (14)
N3	0.0439 (14)	0.0479 (16)	0.0434 (15)	0.0199 (12)	0.0110 (11)	0.0097 (12)
N4	0.0469 (15)	0.0542 (17)	0.0562 (17)	0.0202 (13)	0.0243 (13)	0.0174 (14)
O1	0.0535 (15)	0.110 (2)	0.0619 (19)	0.0426 (16)	0.0251 (13)	0.0259 (18)
O2	0.0739 (19)	0.064 (2)	0.123 (3)	0.0204 (16)	0.064 (2)	0.026 (2)
O3	0.0821 (19)	0.0466 (15)	0.0721 (18)	0.0207 (14)	0.0283 (15)	0.0095 (14)
O4	0.094 (2)	0.0665 (19)	0.092 (2)	0.0296 (17)	0.0422 (19)	0.0197 (18)
O5	0.0527 (15)	0.088 (2)	0.0659 (18)	0.0314 (14)	0.0095 (12)	0.0301 (16)
O6	0.0664 (18)	0.136 (3)	0.0616 (18)	0.0385 (19)	0.0151 (15)	0.038 (2)
Cd1	0.04362 (14)	0.04697 (15)	0.04136 (15)	0.02141 (11)	0.01461 (9)	0.01174 (10)

### Geometric parameters (Å, º)

**Table:**

C1—N1	1.335 (5)	C12—N4	1.254 (5)
C1—C2	1.379 (6)	C12—H12A	0.9300
C1—H1	0.9300	C13—C14	1.502 (6)
C2—C3	1.357 (7)	C13—H13A	0.9600
C2—H2	0.9300	C13—H13B	0.9600
C3—C4	1.378 (7)	C13—H13C	0.9600
C3—H3	0.9300	C14—O4	1.235 (5)
C4—C5	1.377 (6)	C14—O3	1.246 (5)
C4—H4	0.9300	C15—C16	1.499 (5)
C5—N1	1.342 (5)	C15—H15A	0.9600
C5—C6	1.472 (5)	C15—H15B	0.9600
C6—N2	1.263 (5)	C15—H15C	0.9600
C6—H6A	0.9300	C16—O6	1.237 (5)
C7—N3	1.330 (5)	C16—O5	1.248 (4)
C7—C8	1.384 (6)	N2—O1	1.373 (4)
C7—H7	0.9300	N4—O2	1.367 (4)
C8—C9	1.366 (6)	O1—H1B	0.830 (10)
C8—H8	0.9300	O2—H2B	0.83 (5)
C9—C10	1.359 (6)	Cd1—N1	2.438 (3)
C9—H9	0.9300	Cd1—N2	2.362 (3)
C10—C11	1.376 (5)	Cd1—N3	2.411 (3)
C10—H10	0.9300	Cd1—N4	2.344 (3)
C11—N3	1.346 (4)	Cd1—O3	2.245 (3)
C11—C12	1.468 (6)	Cd1—O5	2.210 (3)
			
N1—C1—C2	122.3 (4)	O4—C14—C13	117.2 (4)
N1—C1—H1	118.8	O3—C14—C13	117.3 (4)
C2—C1—H1	118.8	C16—C15—H15A	109.5
C3—C2—C1	119.3 (4)	C16—C15—H15B	109.5
C3—C2—H2	120.3	H15A—C15—H15B	109.5
C1—C2—H2	120.3	C16—C15—H15C	109.5
C2—C3—C4	119.4 (4)	H15A—C15—H15C	109.5
C2—C3—H3	120.3	H15B—C15—H15C	109.5
C4—C3—H3	120.3	O6—C16—O5	124.8 (4)
C5—C4—C3	118.6 (4)	O6—C16—C15	118.3 (3)
C5—C4—H4	120.7	O5—C16—C15	116.9 (4)
C3—C4—H4	120.7	C1—N1—C5	118.0 (3)
N1—C5—C4	122.4 (4)	C1—N1—Cd1	127.0 (2)
N1—C5—C6	116.9 (3)	C5—N1—Cd1	115.0 (2)
C4—C5—C6	120.7 (4)	C6—N2—O1	115.0 (3)
N2—C6—C5	119.1 (3)	C6—N2—Cd1	118.9 (2)
N2—C6—H6A	120.4	O1—N2—Cd1	124.7 (2)
C5—C6—H6A	120.4	C7—N3—C11	117.1 (3)
N3—C7—C8	123.4 (4)	C7—N3—Cd1	127.8 (2)
N3—C7—H7	118.3	C11—N3—Cd1	115.0 (2)
C8—C7—H7	118.3	C12—N4—O2	115.3 (3)
C9—C8—C7	118.3 (4)	C12—N4—Cd1	118.4 (2)
C9—C8—H8	120.8	O2—N4—Cd1	126.3 (2)
C7—C8—H8	120.8	N2—O1—H1B	103 (3)
C10—C9—C8	119.2 (4)	N4—O2—H2B	109 (4)
C10—C9—H9	120.4	C14—O3—Cd1	129.1 (3)
C8—C9—H9	120.4	C16—O5—Cd1	124.6 (3)
C9—C10—C11	119.7 (4)	O5—Cd1—O3	95.55 (12)
C9—C10—H10	120.1	O5—Cd1—N4	96.42 (11)
C11—C10—H10	120.1	O3—Cd1—N4	100.57 (11)
N3—C11—C10	122.1 (4)	O5—Cd1—N2	103.25 (10)
N3—C11—C12	116.3 (3)	O3—Cd1—N2	91.65 (11)
C10—C11—C12	121.5 (3)	N4—Cd1—N2	155.72 (12)
N4—C12—C11	120.8 (3)	O5—Cd1—N3	92.09 (11)
N4—C12—H12A	119.6	O3—Cd1—N3	168.11 (10)
C11—C12—H12A	119.6	N4—Cd1—N3	69.45 (10)
C14—C13—H13A	109.5	N2—Cd1—N3	95.46 (10)
C14—C13—H13B	109.5	O5—Cd1—N1	170.71 (10)
H13A—C13—H13B	109.5	O3—Cd1—N1	89.02 (11)
C14—C13—H13C	109.5	N4—Cd1—N1	90.68 (10)
H13A—C13—H13C	109.5	N2—Cd1—N1	68.45 (10)
H13B—C13—H13C	109.5	N3—Cd1—N1	84.77 (10)
O4—C14—O3	125.5 (4)		
			
N1—C1—C2—C3	−1.5 (7)	C14—O3—Cd1—N3	43.8 (7)
C1—C2—C3—C4	1.0 (8)	C14—O3—Cd1—N1	102.2 (4)
C2—C3—C4—C5	0.8 (8)	C12—N4—Cd1—O5	−92.2 (3)
C3—C4—C5—N1	−2.3 (7)	O2—N4—Cd1—O5	88.5 (3)
C3—C4—C5—C6	177.3 (4)	C12—N4—Cd1—O3	170.9 (3)
N1—C5—C6—N2	7.4 (6)	O2—N4—Cd1—O3	−8.4 (3)
C4—C5—C6—N2	−172.3 (4)	C12—N4—Cd1—N2	51.9 (4)
N3—C7—C8—C9	−0.9 (6)	O2—N4—Cd1—N2	−127.4 (4)
C7—C8—C9—C10	−0.4 (7)	C12—N4—Cd1—N3	−2.4 (3)
C8—C9—C10—C11	1.4 (7)	O2—N4—Cd1—N3	178.3 (4)
C9—C10—C11—N3	−1.2 (7)	C12—N4—Cd1—N1	81.8 (3)
C9—C10—C11—C12	178.3 (4)	O2—N4—Cd1—N1	−97.5 (3)
N3—C11—C12—N4	0.6 (6)	C6—N2—Cd1—O5	−172.9 (3)
C10—C11—C12—N4	−178.9 (4)	O1—N2—Cd1—O5	−7.2 (3)
C2—C1—N1—C5	0.1 (6)	C6—N2—Cd1—O3	−76.8 (3)
C2—C1—N1—Cd1	179.1 (3)	O1—N2—Cd1—O3	88.9 (3)
C4—C5—N1—C1	1.9 (6)	C6—N2—Cd1—N4	43.8 (5)
C6—C5—N1—C1	−177.8 (3)	O1—N2—Cd1—N4	−150.5 (3)
C4—C5—N1—Cd1	−177.3 (4)	C6—N2—Cd1—N3	93.6 (3)
C6—C5—N1—Cd1	3.0 (4)	O1—N2—Cd1—N3	−100.7 (3)
C5—C6—N2—O1	178.4 (3)	C6—N2—Cd1—N1	11.5 (3)
C5—C6—N2—Cd1	−14.5 (5)	O1—N2—Cd1—N1	177.2 (3)
C8—C7—N3—C11	1.1 (5)	C7—N3—Cd1—O5	−84.9 (3)
C8—C7—N3—Cd1	−175.4 (3)	C11—N3—Cd1—O5	98.6 (2)
C10—C11—N3—C7	0.0 (5)	C7—N3—Cd1—O3	145.1 (5)
C12—C11—N3—C7	−179.5 (3)	C11—N3—Cd1—O3	−31.4 (6)
C10—C11—N3—Cd1	176.9 (3)	C7—N3—Cd1—N4	179.1 (3)
C12—C11—N3—Cd1	−2.6 (4)	C11—N3—Cd1—N4	2.6 (2)
C11—C12—N4—O2	−178.7 (4)	C7—N3—Cd1—N2	18.7 (3)
C11—C12—N4—Cd1	2.0 (5)	C11—N3—Cd1—N2	−157.8 (2)
O4—C14—O3—Cd1	−6.0 (6)	C7—N3—Cd1—N1	86.4 (3)
C13—C14—O3—Cd1	175.2 (3)	C11—N3—Cd1—N1	−90.1 (2)
O6—C16—O5—Cd1	6.3 (6)	C1—N1—Cd1—O5	146.5 (6)
C15—C16—O5—Cd1	−172.3 (3)	C5—N1—Cd1—O5	−34.4 (7)
C16—O5—Cd1—O3	−111.5 (3)	C1—N1—Cd1—O3	−93.9 (3)
C16—O5—Cd1—N4	147.2 (3)	C5—N1—Cd1—O3	85.2 (3)
C16—O5—Cd1—N2	−18.5 (3)	C1—N1—Cd1—N4	6.7 (3)
C16—O5—Cd1—N3	77.6 (3)	C5—N1—Cd1—N4	−174.3 (3)
C16—O5—Cd1—N1	7.6 (8)	C1—N1—Cd1—N2	173.9 (3)
C14—O3—Cd1—O5	−85.9 (4)	C5—N1—Cd1—N2	−7.0 (3)
C14—O3—Cd1—N4	11.7 (4)	C1—N1—Cd1—N3	76.0 (3)
C14—O3—Cd1—N2	170.6 (3)	C5—N1—Cd1—N3	−105.0 (3)

### Hydrogen-bond geometry (Å, º)

**Table:**

*D*—H···*A*	*D*—H	H···*A*	*D*···*A*	*D*—H···*A*
O1—H1*B*···O6	0.83 (2)	1.72 (1)	2.545 (4)	169 (4)
O2—H2*B*···O4	0.83 (5)	1.69 (5)	2.512 (5)	175 (6)
C6—H6*A*···O3^i^	0.93	2.52	3.381 (5)	153
C9—H9···O5^ii^	0.93	2.55	3.387 (6)	151
C12—H12*A*···O6^iii^	0.93	2.56	3.264 (6)	132

Symmetry codes: (i) −*x*+1, −*y*+2, −*z*+2; (ii) *x*, *y*−1, *z*; (iii) *x*+1, *y*, *z*.
